# Retroperitoneal Malignant Fibrous Histiocytoma Can Mimic a Hydatid Cyst

**DOI:** 10.1155/2011/362391

**Published:** 2011-09-21

**Authors:** Gurcan Erbay, Serife Ulusan, Zafer Koc, Emine Tuba Canpolat, Kenan Calıskan

**Affiliations:** ^1^Department of Radiology, Faculty of Medicine, Başkent University, 01250 Adana, Turkey; ^2^Başkent Üniversitesi Adana Hastanesi, Dadaloğlu M., Serin Evler 39, Sokak no: 6, Yüreğir, 01250 Adana, Turkey; ^3^Department of Pathology, Faculty of Medicine, Başkent University, 01250 Adana, Turkey; ^4^Department of General Surgery, Faculty of Medicine, Başkent University, 01250 Adana, Turkey

## Abstract

Malignant fibrous histiocytoma is the second most common soft-tissue sarcoma in adults. After the extremities, the retroperitoneal space is the second most common site of this tumor. A 50-year-old man presented with a right retroperitoneal, thick-walled, cystic multilocular mass measuring 10 × 10 cm that was thought to be a type CE 5 hydatid cyst preoperatively. However, the postoperative histopathology did not agree with the radiological findings and instead showed a malignant fibrous histiocytoma. The computed tomography and ultrasound/Doppler ultrasound findings of this retroperitoneal mass mimicked a type CE 5 hydatid cyst. We present this case because the surgical management of these two lesions differs and misdiagnosis can be problematic.

## 1. Introduction

Ozello et al. first described malignant fibrous histiocytoma (MFH) in 1963 [1]. A MFH is a soft-tissue sarcoma of undifferentiated mesenchymal cell origin. MFH has an atypical origin in the subcutis and deep dermal layers [2]. It is the second most common soft-tissue sarcoma in adults. It commonly occurs in the 5th and 6th decades, with a 2 : 1 male-to-female predominance. After the extremities, the retroperitoneal space is the second most common site of this tumor [3, 4]. Because the radiological findings of MFH are nonspecific, an accurate diagnosis of retroperitoneal MFH is difficult. We report a rare case of retroperitoneal MFH that was misdiagnosed radiologically as a type CE 5 hydatid cyst preoperatively.

## 2. Case

**Figure 1:**
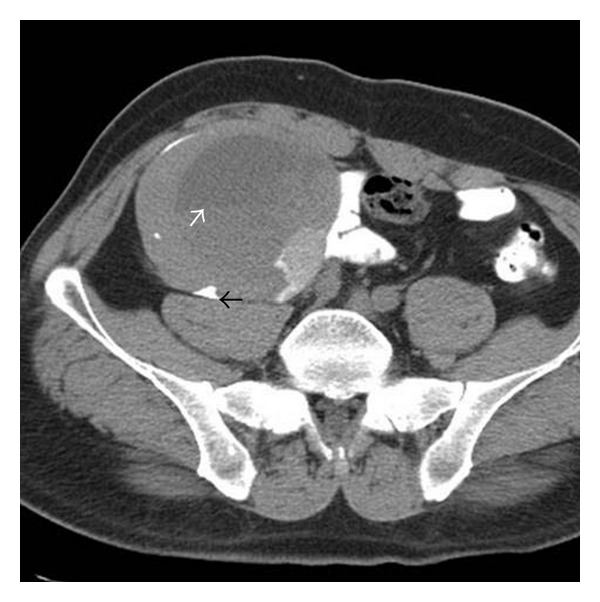
Abdominal precontrast CT shows a thick-walled, cystic, multilocular mass, measuring 10 × 10 cm in front of the right psoas and iliopsoas muscles and adjacent to the iliac vessels. Spotty calcification (black arrow) was seen in the thick, hyperdense wall. White arrow shows fluid-fluid levels.

**Figure 2:**
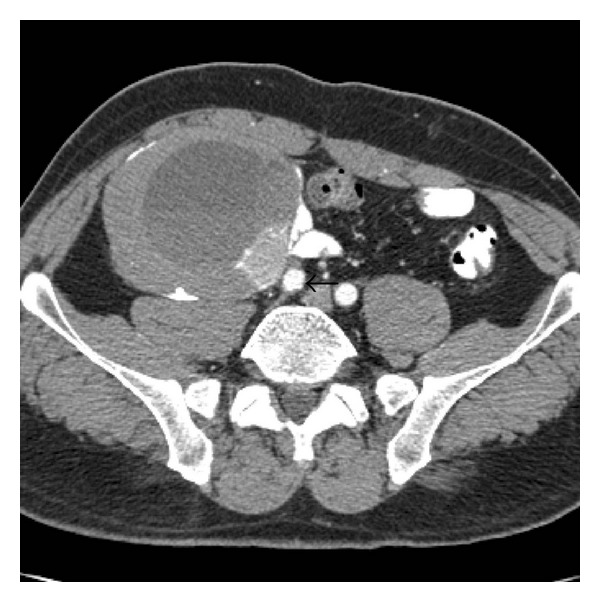
On enhanced CT, the wall and septa were not enhanced.

A 50-year-old man presented with right leg and low-back pain. His medical history included a cholecystectomy and essential hypertension. On physical examination, a fixed hard mass was palpated in the right lower abdominal quadrant. Laboratory results were normal, including CEA and CA 19–9. Abdominal computed tomography (CT) revealed a thick-walled, cystic, multilocular mass measuring 10 × 10 cm in front of the right psoas and iliopsoas muscles and adjacent to the iliac vessels. Spotty calcification was seen in the thick, hyperdense wall on nonenhanced CT (Figure 1). On enhanced CT the wall and septae were not enhanced (Figure 2). On Doppler ultrasound (US), no vascular mapping was seen in the wall or septae, but fluid-fluid levels were seen. Based on the US, Doppler US, and pre- and postcontrast abdominal CT findings, a retroperitoneal type CE5 hydatid cyst was diagnosed preoperatively.

At surgery, the retroperitoneal mass was successfully resected en bloc without damaging the right iliac vein or artery. Chest CT to look for possible lung metastases was performed after MFH was diagnosed histopathologically. Chemotherapy was not planned. Adjuvant radiotherapy to the retroperitoneal region was recommended.

**Figure 3:**
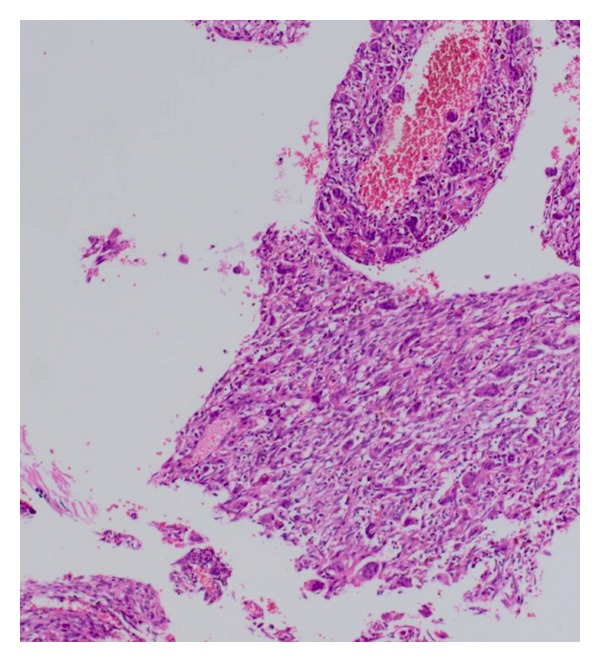
The giant cell form of malignant fibrous histiocytoma contains less mature bone in the tumor (H and E × 200).

**Figure 4:**
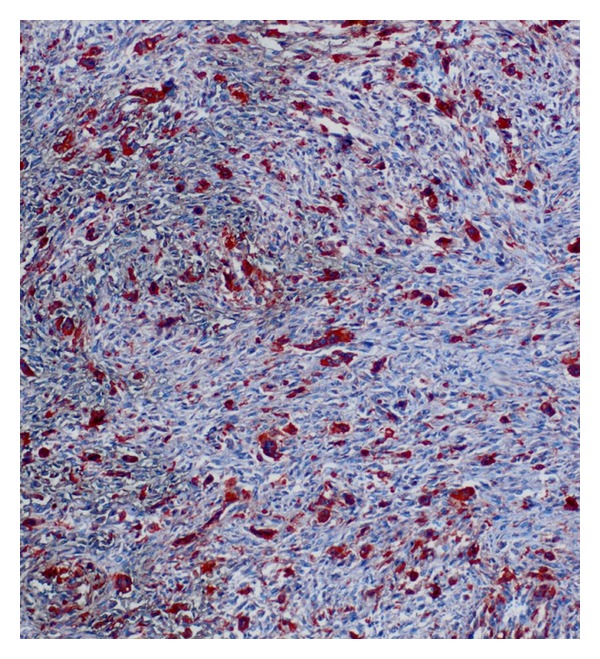
Immunohistochemical CD68 staining of the malignant fibrous histiocytoma (×400).

**Figure 5:**
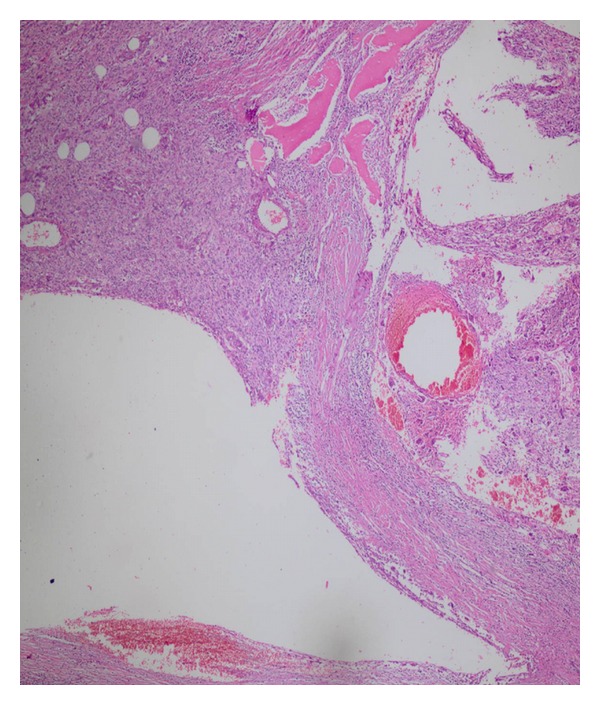
Hemorrhagic areas and aneurismal bone cyst-like areas.

Macroscopically, the postoperative pathologic examination showed that the tumor was a 12 × 10 × 9 cm solid mass, with a thin calcified capsule. Microscopically, the tumor contained spindle cells and fibroblast-like cells arranged in short fascicles and pleomorphic bizarre histiocyte-like cells, admixed with osteoclast-like multinucleate cells. There was an aneurismal bone cyst-appearing hemorrhagic area, with focal osteoid and mature bone in the tumor capsule (Figures 3 and 4). Immunohistochemically, the tumor cells were positive for CD68 and lysozyme and negative for S-100 and SMA (Figure 5).

## 3. Discussion

Malignant fibrous histiocytoma usually presents as a painless mass with increased intra-abdominal pressure. It can also be accompanied by an increased erythrocyte sedimentation rate, weight loss, and fever. MFH originates from undifferentiated mesenchymal cells [3] and can be a complication of radiation, chronic postoperative repair, trauma, surgical incisions or burn scars, and previously diagnosed hematopoietic disease, including Hodgkin's lymphoma, multiple melanoma, and malignant histiocytosis [2, 3].

Malignant fibrous histiocytomas are tumors with a mixed structure, containing fibroblasts and histiocyte-like cells, to varying degrees. The tumor tissue may also comprise tumor giant cells, inflammatory cells, and xanthoma cells. The tumor may be interspersed with myxoid substance and elastic fibers [5]. 

Five histological subtypes of MFH have been described: pleomorphic storiform (65%), myxoid (15%), giant cell (10%), inflammatory (8%), and angiomatoid (2%). The histological differential diagnosis of MFH includes leiomyosarcoma, liposarcoma, and rhabdomyosarcoma. MFH is most frequent in those older than 70 years, and it rarely occurs under the age of 40 [5]. 

The prognosis of MFH is related to the histological malignity grade and the tumor size, depth, and location [3]. Bertoni et al. observed recurrence in 37.5% of cases. The 5-year survival rate was 36% according to Pezzi et al. while Enziger et al. observed recurrence in 25% and metastasis in 34%, with a recovery rate of 50% [5].

Our findings suggest that the incidence of dedifferentiated liposarcoma is underestimated and that many retroperitoneal liposarcomas and MFHs are dedifferentiated liposarcomas [6]. Retroperitoneal MFHs are rare tumors that are difficult to diagnose preoperatively. The radiological findings are usually not diagnostic. Radical surgery is recommended for the treatment of MFH. Radiotherapy and cytostatic therapy are also used, alone or palliatively [3]. 

Although CT and magnetic resonance imaging (MRI) can demonstrate important characteristics of these tumors, the diagnosis is often challenging for radiologists. Diagnostic challenges include the precise localization of the lesion, determination of the extent of invasion, and characterization of the specific pathological type. Some radiological signs that are helpful in determining tumor origin include the beak, phantom (invisible) organ, embedded organ, and prominent feeding artery signs. In our case, CT showed the true location of the tumor and invasion of the adjacent vessels and neighboring organs. 

When there is no definite sign that suggests the organ of origin, the diagnosis of primary retroperitoneal tumor is likely [7]. CT shows MFH as a large, lobulated, soft-tissue mass, with attenuation similar to that of muscle. Frequently, areas of decreased attenuation are apparent more centrally within the mass, corresponding to myxoid regions, hemorrhage, or necrosis. Enhancement of the solid portions of MFH, which is often nodular and peripheral, is seen on CT following the administration of intravenous contrast. Focal or diffuse, calcification is seen in approximately 10% of retroperitoneal MFH and is usually coarse in appearance. Hemorrhagic components are common in soft tissue MFH. These are usually recognized on CT as areas of increased attenuation. Fluid-fluid levels may be seen on CT or MRI after hemorrhage, representing sedimentation of blood products [4]. Sonography reveals a hypoechoic solid mass, although occasionally it has a more heterogeneous appearance. This radiological finding is certainly nonspecific.

In our case, CT and US showed a large, well-defined, soft-tissue mass with areas of decreased attenuation and focal and diffuse calcification. In contrast to the reported radiological findings, there was no marked enhancement.

According to the World Health Organization informal working group, the international classification of ultrasound images in type CE5 cystic echinococcosis is characterized by a thick calcified wall, which is arch-shaped, producing a cone-shaped shadow. The degree of calcification ranges from partial to complete. These features are not pathognomonic but are suggestive of Echinococcus granulosus [8].

The estimated frequency of primary retroperitoneal hydatid cysts is 0.8–1% in the-English language literature. The cyst is located in the right retroperitoneum in 50% of cases, the left retroperitoneum in 35.7%, the perivesicular region in 7.1%, and the paravesicular region in 7.1%. Symptoms included flank pain in 57.1% of the patients and a palpable mass in 42.8%. Types CE1 to CE5 cysts are seen in 21.4, 37.5, 21.4, 14.3, and 7.2% of the patients, respectively [9]. 

We misdiagnosed this tumor as a type CE5 hydatid cyst preoperatively because it was a large cystic mass with a calcified wall in a retroperitoneal location. After careful review of the patient's clinical data and CT and Doppler US imaging findings, we found a cystic mass containing myxoid stroma and fluid-fluid levels. These radiologic findings narrowed the differential diagnosis, and the possibility of MFH should have been considered.

In conclusion, retroperitoneal MFHs are rare tumors that are difficult to diagnose preoperatively. The radiologic findings are usually nonspecific and are not diagnostic. The treatment of choice is complete radical surgical excision, followed by radiotherapy or hypostatic chemotherapy.

Histopathologically, the microscopic examination must differentiate MFH from aneurysmal fibrous histiocytoma and extraosseous osteosarcoma. Large blood-filled spaces, giant cells, and mature osteoid metaplasia in the capsule were seen in our case. An aneurysmal fibrous histiocytoma is a rare variant of cutaneous fibrous histiocytoma that results from blood vessel proliferation and hemorrhage into a fibrous histiocytoma. Its morphology is similar to MFH. These lesions contain abundant hemosiderin and mitotic activity near the hemorrhagic area. Osteoid metaplasia and mature bone are not present and the tumor always has a subcutaneous location. The giant cell form of MFH contains less mature bone. The osteoid is relatively focal and mature; if osteoid is predominant, the diagnosis is extra-osseous osteosarcoma [10]; our case contained mature bone.

The thick lamellar osseous tissue with a thick fibrous, calcified wall and aneurismal bone with cystic areas was unique to our case. The histopathological type was the angiomatoid type. Its morphological features might mimic a hydatid cyst radiologically.
